# Supplementary material for: Federated learning based futuristic biomedical big-data analysis and standardization
Source: PLoS One. 2023 Oct 4;18(10):e0291631. doi: 10.1371/journal.pone.0291631 (PMC10550167; doi:10.1371/journal.pone.0291631)
Supplement: Attachment — Submitted filename: Reviewer response.docx [file pone.0291631.s001.docx]

**Reviewer 1:**

[1] The abstract should end with a brief statement regarding the significance and impact of this paper.

**Response:** We have added the accuracy and correlated performance matrix

[2] The method of comparison experiment used in the manuscript is too old. I have not found the recent works published in the year 2022-2023. The following manuscripts can be added.

- H. Manoharan, S. Selvarajan, A. Yafoz, H. A. Alterazi, and C. Chen, “Deep Conviction Systems for Biomedical Applications Using Intuiting Procedures With Cross Point Approach,” vol. 10, no. May, pp. 1–14, 2022, doi: 10.3389/fpubh.2022.909628.
- S. Selvarajan, H. Manoharan, T. Hasanin, R. Alsini, and M. Uddin, “applied sciences Biomedical Signals for Healthcare Using Hadoop Infrastructure with Artificial Intelligence and Fuzzy Logic Interpretation,” 2022.
- H. Manoharan et al., “A machine learning algorithm for classification of mental tasks,” Comput. Electr. Eng., vol. 99, no. February, p. 107785, 2022, doi: 10.1016/j.compeleceng.2022.107785.
- P. R. Kshirsagar, H. Manoharan, S. Shitharth, A. M. Alshareef, N. Albishry, and P. K. Balachandran, “Deep Learning Approaches for Prognosis of Automated Skin Disease,” Life, vol. 12, no. 3, p. 426, 2022, doi: 10.3390/life12030426.
- A. O. Khadidos, H. Manoharan, S. Selvarajan, and A. O. Khadidos, “A Classy Multifacet Clustering and Fused Optimization Based Classification Methodologies for SCADA Security,” pp. 1–24, 2022.
- S. Shitharth, P. Meshram, P. R. Kshirsagar, H. Manoharan, V. Tirth, and V. P. Sundramurthy, “Impact of Big Data Analysis on Nanosensors for Applied Sciences Using Neural Networks,” J. Nanomater., vol. 2021, 2021, doi: 10.1155/2021/4927607.

**Response:** We have included the above references.

[3] Abstract should be shortened appropriately. Please reduce the content of the abstract and highlight the merits of the proposed scheme.

**Response:** Updated

[4] The manuscript lacks evaluation indicators, please add several evaluation indicators for further comparative analysis.

**Response:** We have updated the comparative analysis models in mathematical section under Table. 1

[5] The conclusions should explain the comparative results between the proposed and state-of-the-art methods.

**Response:** Performance metric and comparative explanation is added in conclusion.

[6] The work is well written and well-presented but needs to be proofread in English as it has some typos.

**Response:** English and Typo check is performed

[7] Although the authors provide a contextualization of the problem in the introduction, it is not clear what the contribution of the article is.

**Response:** The introduction is rewritten and contribution of article with relevance to the proposed work and filed is highlighted.

**Reviewer 2:** The authors have done a good job on the Federated Learning-based Futuristic Biomedical Big-data Analysis, but this paper still needs improvement.

1. The abstract should always mention the rate of efficacy/efficiency percentage of the proposed method for the reader’s quick overview.

**Response:** We have added the performance metric

2. The abstract should at least have a line or two about the need for this work. This abstract has an intro and it straightaway deals with the proposed work.

**Response:** The entire, abstract is rewritten and the comments incorporated.

3. Apart from giving the literature in paragraphs, better to add a table which precisely shows the proposed method, pros and cons.

**Response:** The comparative parameters on the study is limited, hence we could not provide a table format of pros and cons

4. The novelty of this paper is not clear. The difference between the present work and previous works should be highlighted. Add more of the issues and what is the significance of this research.

**Response:** This comment is highlighted in Introduction section

5. The paper lacks a convincing theoretical framework, which is necessary to be considered for publication.

**Response:** Theoretical explanation is added and enhanced in the section 3

6. Any research paper should include a separate objective section so that readers can easily see the article’s goal. Authors ought to create a separate section for it.

**Response:** The objective is added in Introduction, the same is highlighted for your kind consideration.

7. Though the paper is mathematically strong, nomenclature should be included. It’s very difficult to follow up on the equations.

**Response:** We tried our best to optimize the equations in revised

8. The use of more parametric comparisons by the authors is recommended. Confusion matrices are a key component for any system’s validity. However, they are seldom mentioned by the authors.

**Response:** Table. 1 discussed on these parameters.

9. Another major issue of this paper is missing implementation details. The authors haven't mentioned anything about the tool used to build/implement the algorithm. The language they used, and where is the sample code of that? If not at least there should be algorithm’s pseudocode.

**Response:** The implementation details are added in results and discussion section.

10. The references are very less for a scientific article. Among 10 references, not even a single reference is from 2022 and none from 2023.

**Response:** We have updated our reference section

11. Authors are advised to follow the IMRAD format for the entire paper.

**Response:** We have assured the paper is in IMRAD format

12. A separate section for Limitations and future work in detail would give further ideas for the readers who wish to enhance your work.

**Response:** We have considered to keep it in conclusion.
